# MBSP1: a biosurfactant protein derived from a metagenomic library with activity in oil degradation

**DOI:** 10.1038/s41598-020-58330-x

**Published:** 2020-01-28

**Authors:** Sinara Carla da Silva Araújo, Rita C. B. Silva-Portela, Daniel Chaves de Lima, Marbella Maria Bernardes da Fonsêca, Wydemberg J. Araújo, Uaska Bezerra da Silva, Amanda P. Napp, Evandro Pereira, Marilene H. Vainstein, Lucymara Fassarella Agnez-Lima

**Affiliations:** 1 Department of Cellular Biology and Genetics, Universidade Federal do Rio Grande do Norte, Natal, Rio Grande do Norte Brazil; 2 Center of Biotechnology, Universidade Federal do Rio Grande do Sul, Porto Alegre, Rio Grande do Sul Brazil

**Keywords:** Archaeal genes, Bioremediation

## Abstract

Microorganisms represent the most abundant biomass on the planet; however, because of several cultivation technique limitations, most of this genetic patrimony has been inaccessible. Due to the advent of metagenomic methodologies, such limitations have been overcome. Prevailing over these limitations enabled the genetic pool of non-cultivable microorganisms to be exploited for improvements in the development of biotechnological products. By utilising a metagenomic approach, we identified a new gene related to biosurfactant production and hydrocarbon degradation. Environmental DNA was extracted from soil samples collected on the banks of the Jundiaí River (Natal, Brazil), and a metagenomic library was constructed. Functional screening identified the clone 3C6, which was positive for the biosurfactant protein and revealed an open reading frame (ORF) with high similarity to sequences encoding a hypothetical protein from species of the family *Halobacteriaceae*. This protein was purified and exhibited biosurfactant activity. Due to these properties, this protein was named metagenomic biosurfactant protein 1 (MBSP1). In addition, *E. coli Rosetta*^TM^ (DE3) strain cells transformed with the MBSP1 clone showed an increase in aliphatic hydrocarbon degradation. In this study, we described a single gene encoding a protein with marked tensoactive properties that can be produced in a host cell, such as *Escherichia coli*, without substrate dependence. Furthermore, MBSP1 has been demonstrated as the first protein with these characteristics described in the Archaea or Bacteria domains.

## Introduction

Surfactants are amphipathic compounds that have a hydrophobic moiety that is directed towards the surface and a hydrophilic portion that is directed towards the solution^1^. These amphiphilic molecules can reduce surface tension at air/water and oil/water interfaces^2,3^. Surfactants produced by organisms are called biosurfactants, which are extracellular products or components within the cell membranes of prokaryotes and eukaryotes^1,4,5^. Biosurfactants are classified into four major categories: glycolipids, fatty acids, lipopeptides, and polymeric types. These categories are represented by amphipathic polysaccharides, lipopolysaccharides, lipoproteins, fatty acids, or complex mixtures of these biopolymers. In general, the synthesis of biosurfactants involves elaborate genetic systems, including operons, non-ribosomal peptide synthetases, and/or multiprotein assembly complexes^6–9^.

The synthesis of biosurfactants occurs in the presence of different substrates as a carbon source. To reduce production costs, cheaper substrates have been used. The most commonly used substrates for biosurfactant production are agro-industrial products such as molasses, marc, or vegetable oils^6–8^. Rhamnolipids and surfactin are among the best studied biosurfactants. Rhamnolipids are glycolipids first discovered in *Pseudomonas aeruginosa*, which are formed by the bonding between a rhamnose moiety and a 3-(3-hydroxyalkanoyloxy)alkanoic acid (HAA) fatty acid tail. The essential enzymes involved in this pathway are RhlA, RhlB, and RhlC, which are under quorum sensing control. RhlA catalyses the formation of HAA using β-hydroxydecanoyl-ACP as a precursor. RhlB is a rhamnosyltransferase that catalyses the bond between HAA and dTDP-L-rhamnose, resulting in the formation of mono-rhamnolipid. The enzyme RhlC adds a second rhamnose moiety to mono-rhamnolipids forming di-rhamnolipids^10^.

Surfactin is a cyclic lipopeptide discovered in *Bacillus* sp. It is a heptapeptide attached to a β-hydroxy fatty acid chain forming a cyclic lactone ring structure. The synthesis of surfactin is accomplished by a nonribosomal peptide (NRP) synthetases system, encoded by the *srfA* operon, which contains three genes (*srfA, srfB*, and *srfC*), controlled by quorum sensing system^6,9^.

Biosurfactants have several biotechnological properties; for example, biosurfactants are capable of reducing surface and interfacial tension. Additionally, biosurfactant properties have been shown to exhibit emulsification, de-emulsification, dispersion, solubilisation, and mobilisation. These properties permit the use of biosurfactants in the environmental field for hydrocarbon biodegradation and bioremediation. To date, the largest market for biosurfactants is the oil industry, mainly due to its wide array of applications such as bioremediation and dispersion of oil spills, removal and mobilisation of oil residues in storage tanks, and improved oil recovery. Nevertheless, biosurfactant applications in other industries, such as pharmaceutical, cosmetic, and food, are broadly dispersed^2,11–14^.

Biosurfactants offer many advantages over synthetic surfactants; for example, ecological acceptability due to low toxicity and high biodegradability^15–17^, effectiveness in a wide range of temperatures, stability under extreme conditions (e.g., pH and salinity)^1,18,19^, and higher efficiency than synthetic surfactants^20^. Despite these advantages, the production of biosurfactants at a large scale remains an expensive procedure, in part because of complicated extraction and purification processes, as well as the dependence on suitable substrates for their production^7,8^.

Microorganisms are the primary source of biosurfactants; however, our understanding regarding the diversity of genes and mechanisms related to biosurfactant production is solely based on cultivable microorganisms, which represent less than 1% of the diversity of known microbial species^21^. In this context, metagenomic approaches may demonstrate to be a powerful technology for discovering new enzymes and other valuable biomolecules produced by non-cultivable microorganisms. Specifically, functional screening in metagenomic libraries, which has shown to be useful for discovering new genes since sequence homology is not required for gene identification^22,23^. Despite these advantages, large-scale production of biosurfactants remains to be an expensive procedure, in part because of the complicated extraction and purification processes as well as the dependence on suitable substrates for their production^24^. In this study, we described the identification and characterisation of a new gene, a homolog to a hypothetical protein from the domain Archaea, which represents the first surfactant protein from this domain.

## Results

### Identification of a new gene related to surfactant production

The metagenomic library was obtained from a soil sample from the Jundiaí River (Natal, Brazil), which showed intermittent drainage and salinity reaching four times seawater concentrations. In total, 1,240 clones were screened through a functional selection for the detection of clones with surfactant activity and petroleum degradation. One clone, named 3C6, showed positive results in the drop collapse, emulsification, oil dispersion assays, and hydrocarbon degradation test. Due to these positive results in the functional screening, this clone was selected for functional characterisation described in this study. Sequence analysis of clone 3C6 revealed a 1.4 kb insert containing two open reading frames (ORFs), with 897 and 348 bp, respectively. In this study, we described the functional characterisation of the first ORF (Supplementary Fig. S1). The 897 bp ORF encodes a polypeptide of 298 amino acids with an estimated weight of 31 kDa and a theoretical isoelectric point (pI) of 4.40. This sequence showed a high similarity with hypothetical proteins of the family *Halobacteriaceae*. A total of 20 homologous proteins with 80% or more identity with 3C6 ORF1 were selected, all from organisms belonging to the Halobacterium class. This ORF also showed a 90% identity with a hypothetical protein from *Natrialba taiwanensis* in BLASTP (Supplementary Table S2).

**Figure 1:**
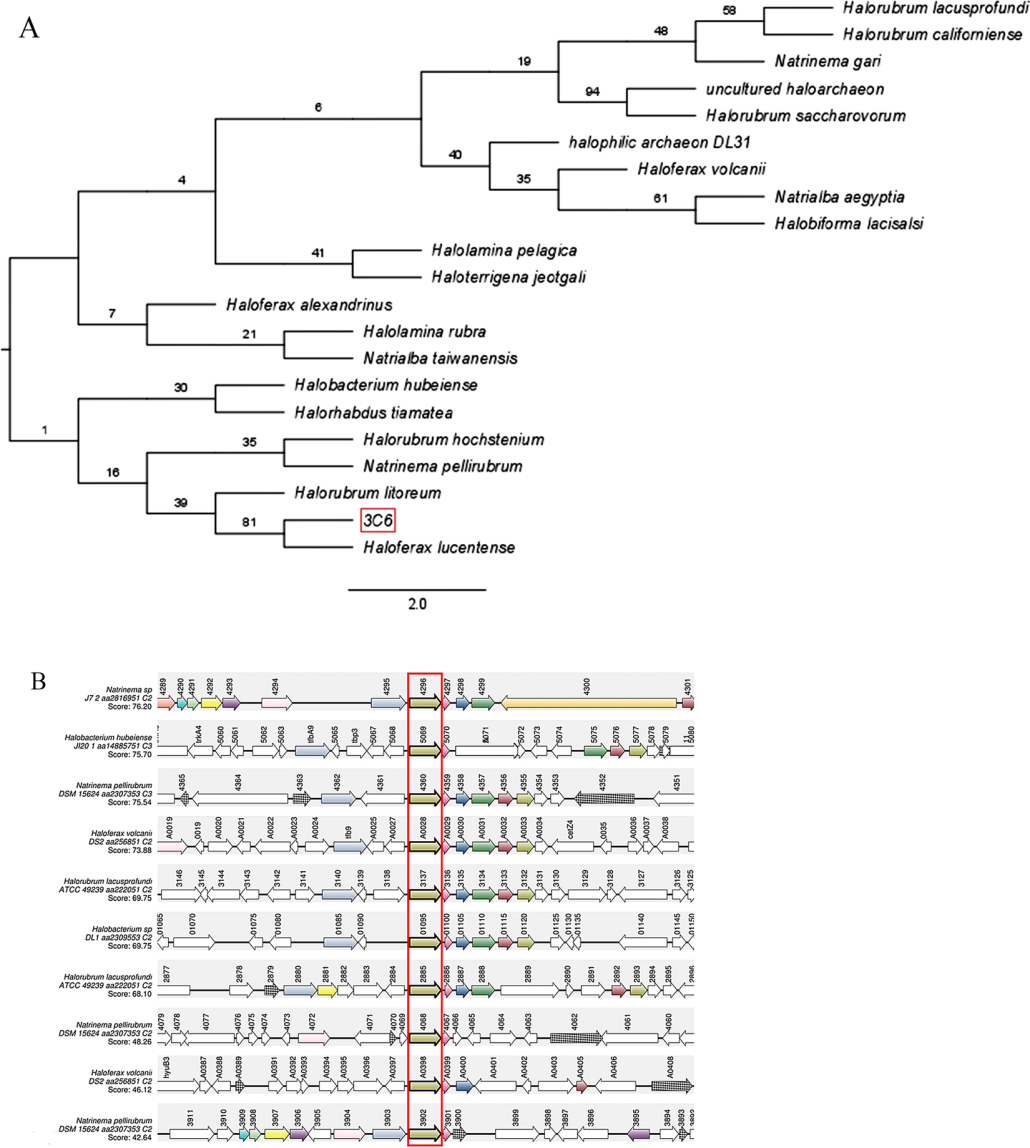
Identification of 3C6 clone (MBSP1): (**A**) Phylogenetic tree obtained from alignment by Neighbour-Joining method highlighting hypothetical protein and highest similarity with *Haloferax lucentense* proteins. The 3C6 ORF is highlighted in the red box; (**B**) Map obtained from Absynte tool displaying the conservation of genes between the species of Archaea. Gene corresponding to 3C6 protein homologs is drawn in red box in centre of map.

Phylogenetic trees showed that 3C6 ORF is indeed related to these hypothetical proteins. This ORF was grouped with *Haloferax lucentense* and *Halorubrum litoreum* in the same branch. The bootstrap analysis demonstrated that this sequence is more similar to *H. lucentense*. The same result was obtained with Neighbour-Joining (Fig. 1A), maximum likelihood, and maximum parsimony methods (Supplementary Fig. S2).

The genomic context of this hypothetical protein was analysed in members of the family *Halobacteriaceae* using Absynte software (Fig. 1B). Synteny of 3C6 homologs were observed in some species. In general, the genomic context shows hypothetical proteins around 3C6 homologs with unknown functions. Since no conserved protein domains were identified in the 3C6 ORF or its homologs, a structural similarity search was performed using predictor servers PHYRE2 and PredictProtein. Data obtained with PHYRE2 showed a very low (non-statistical) similarity with a ribosomal protein (confidence of 70.3) and rubredoxin-like (confidence of 63.4) (Table 1). The PredictProtein software results also indicated structural homology of ORF 3C6 with rubredoxin-like protein, but with low confidence values.

**Table 1:** Data obtained from PHYRE2 showed very low (non-statistical) structural similarity with ribosomal and rubredoxin-like proteins.

Name	Confidence	% i.d.	3C6 ORF 3D model
50S ribosomal protein	70.3	27	
Rubredoxin-like protein	63.4	30	

Using PredictProtein software, it was possible to obtain alignment with 31 proteins from the UniProt database, being all hypothetical proteins from the Archaea domain. The most abundant amino acids in the protein were alanine (13.09%), glycine (9.06%), aspartic acid (9.06%), and glutamic acid (8.05%). Prediction of accessibility to the solvent showed that 62% of residues were wholly exposed, while 32% were buried within the protein core. The physicochemical properties of the amino acid sequence revealed a transmembrane helix located at the 95^th^ residue to the 112^th^, with a size of 18 amino acid residues-long.

**Figure 2:**
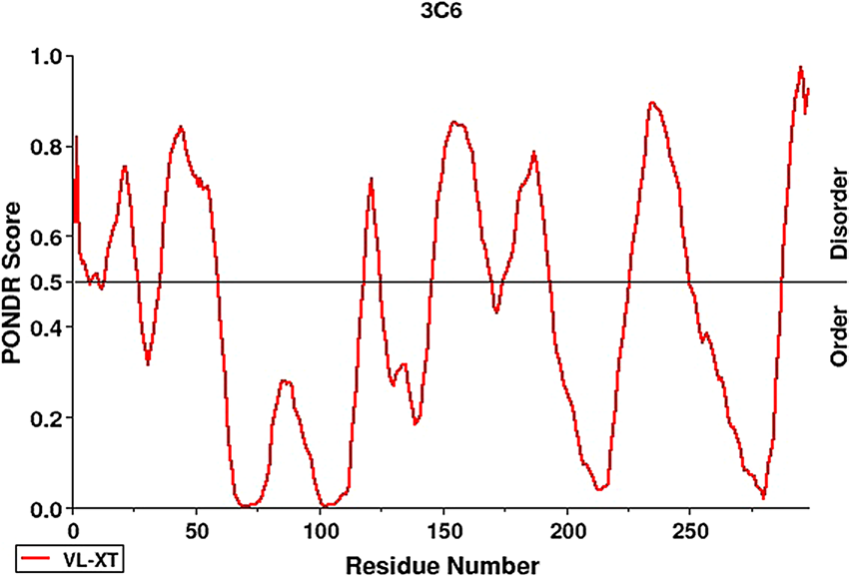
Prediction of intrinsic disorder by PONDR tool. The predicted degree of disorder tendency (scale 0–1; cut-off 0.5) is charted over the amino acid positions within 3C6 ORF (MBSP1). The prediction tool indicates a mostly disordered protein structure. Red bars at the top of the diagram indicate the positions of disordered prediction in the structure.

The prediction of 3C6 subcellular localisation, considering Archaea, Bacteria, and Eukaryote domains, resulted in the following: secreted (100% confidence), periplasm (26% confidence), and nucleus (35% confidence). Disorder prediction using the Predictor of Natural Disordered Regions (PONDR®) tool (Molecular Kinetics, Inc., Indianapolis, IN, USA) showed 131 disordered residues, corresponding to 43.96% of the 3C6 entire structure. These residues are distributed in nine central disordered regions (Fig. 2). The amino acid sequence also showed 36.91% of residues to be hydrophobic, 17.11% acidic, 10.07% basic, and 35.91% neutral.

### 3C6 protein expression

**Figure 3:**
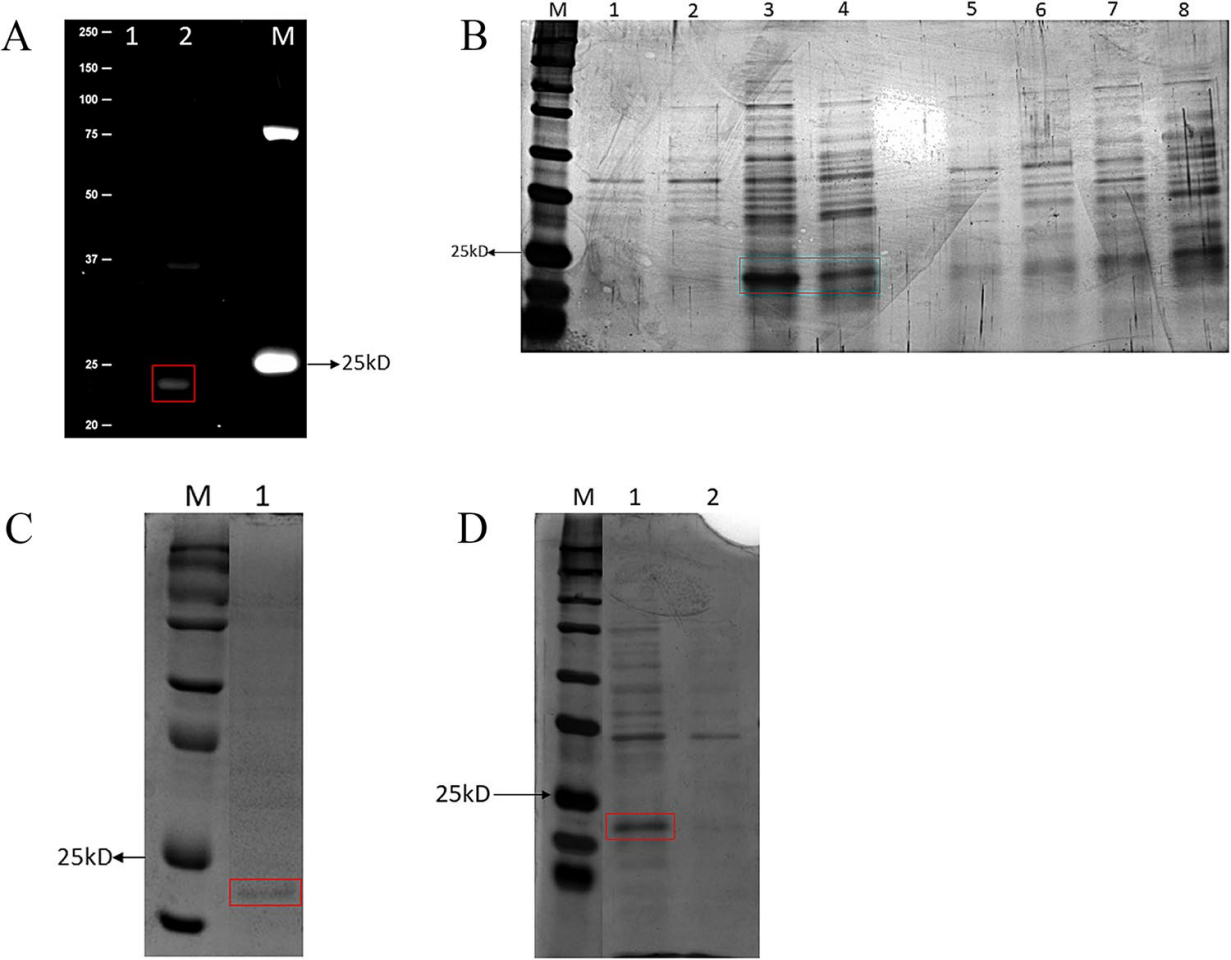
MBSP1 expression: (**A**) Detection of His tagged protein in cell extract using InVision™ His-tag In-gel Stain in polyacrylamide gel electrophoresis (SDS-PAGE). M- *Kaleidoscope* molecular weight marker of 250 kDa (Bio-Rad, Hercules, CA, USA); 1- pHis-parallel1 (negative control). 2- MBSP1 protein (uncropped original picture); (**B**) Analysis of proteins in cell-free supernatant extract using polyacrylamide gel electrophoresis (SDS-PAGE) at 37 °C with different induction times. M - *Kaleidoscope* molecular weight marker of 250 kDa (Bio-Rad, Hercules, CA, USA) 1- MBSP1 clone non-induced. 2- MBSP1 clone induced at 4 h. 3- MBSP1 clone induced at 18 h. 4- MBSP1 without IPTG at 18 h. 5- pHis-parallel1 non-induced. 6- pHis-parallel1 induced for 4 h. 7- pHis-parallel1 induced at 18 h. 8- Non-induced pHis-parallel1 at 18 h (uncropped original picture); (**C**) Protein purified from cell-free supernatant using HisTrap column after ammonium sulfate precipitation with approximate molecular weight of 20 kDa, using polyacrylamide gel electrophoresis (SDS-PAGE). M- *Kaleidoscope* molecular weight marker of 250 kDa (Bio-Rad, Hercules, CA, USA). 1- Purified protein (cropped figure, original gel is shown in Supplementary Fig. S4); (**D**) Proteins after acid precipitation with ammonium sulfate in SDS-PAGE. M- *Kaleidoscope* molecular weight marker of 250 kDa (Bio-Rad, Hercules, CA, USA). 1- Supernatant free of cells carrying MBSP1 after acid precipitation. 2- Supernatant free of cells transformed with pHis-parallel1 (negative control) after acid precipitation (cropped figure, original gel is shown in Supplementary Fig. S5).

The 3C6 ORF was sub-cloned into the pHis-parallel1 expression vector, and cloning was confirmed by enzymatic digestion. The His-tagged protein was detected using InVision™ His-tag In-gel Stain (Invitrogen Corp., Carlsbad, CA, USA) in cell extracts. A protein band with an approximate molecular weight of 20 kDa was detected after induction with IPTG, which was not observed in the empty pHis-parallel1 vector used as the negative control (Fig. 3A). Protein expression was also observed in cell-free supernatants at different induction times (4 h and 18 h) and in the absence of IPTG, indicating that this protein was expressed. A recombinant protein band was visualised at a molecular weight of approximately 20 kDa (Fig. 3B). Cell-free supernatants were precipitated with ammonium sulfate. After precipitation, a protein band was observed in 30–60% fraction and subsequently purified with the HisTrap^TM^ column (GE Healthcare, Chicago, IL, USA), showing a molecular weight around 20 kDa (Fig. 3C). In addition, cell-free supernatants were subjected to surfactant precipitation with acid. A protein of approximately 20 kDa was only detected in the sample from 3C6 cultures (Fig. 3D). We named this protein, “metagenomic biosurfactant protein 1 (MBSP1).”

### MBSP1 biosurfactant activity

**Figure 4:**
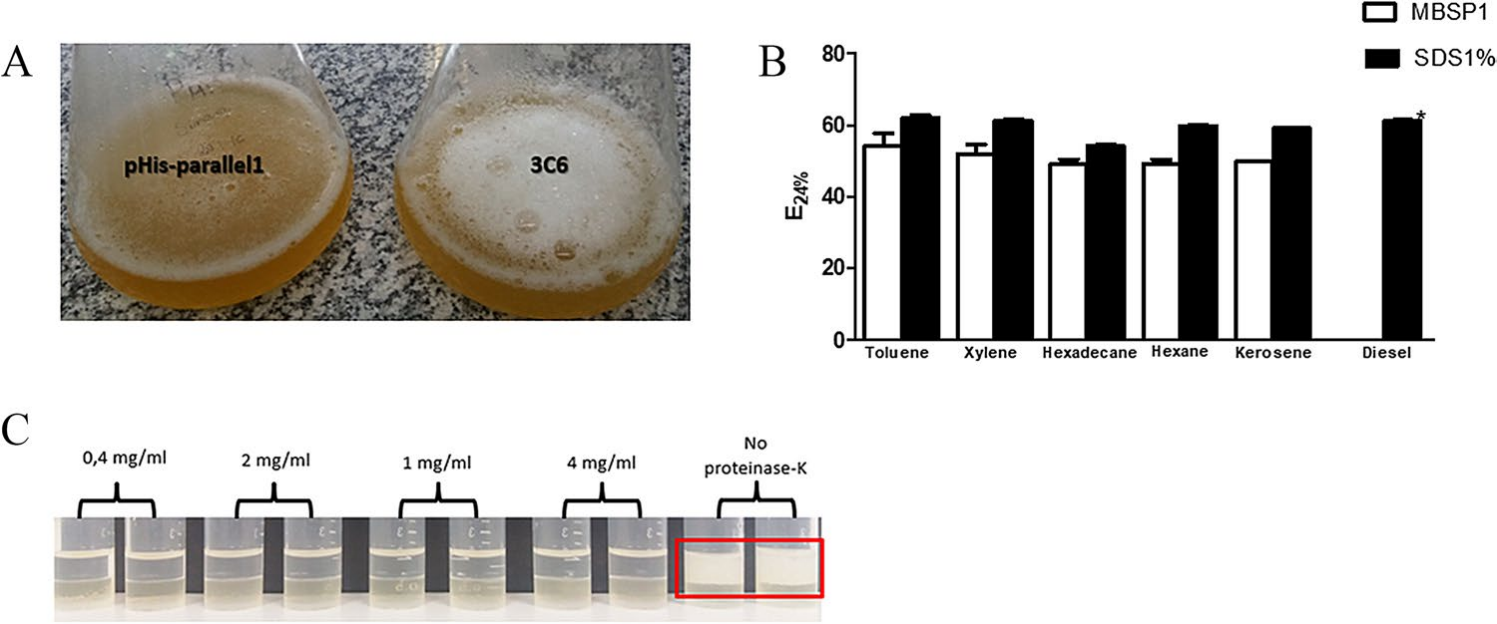
Bacterial culture of clone MBSP1 and empty vector pHis-parallel1 after IPTG induction and emulsification assay with different hydrocarbon sources. (**A**) Emulsion observed in culture containing MBSP1 after induction with IPTG; (**B**) Emulsification index (E24%) showing similarity between MBSP1 and positive control 1% SDS, using different hydrocarbon sources. Statistical difference was only observed in relation to diesel emulsification (*p < 0.05); (**C**) Supernatant containing MBSP1 treated with different concentrations of proteinase K (0.4, 2, 1, 4 mg/ml). In the control, no proteinase K was added, and emulsifying activity was maintained.

Biosurfactant activity was evaluated in cell-free supernatant, purified protein, and surfactant obtained by acid precipitation from bacteria cultures. After induction with IPTG, it was possible to observe the production of emulsion (Fig. 4A). Using cell-free supernatant, emulsification indices were obtained for different substrates. All hydrocarbons tested served as substrates for emulsification, except diesel. Emulsification indices were better in toluene and xylene (56,7% and 51,9%, respectively), followed by hexadecane and hexane (both 49%). Compared with the positive control (1% SDS), only kerosene showed a statistical difference (Fig. 4B). The pHis-parallell empty vector did not show positive emulsification results for any tested sources.

Emulsification was also observed with purified protein and the biosurfactant obtained via acid precipitation (data not shown). The protein nature of the surfactant was confirmed by treatment of the cell-free supernatant with proteinase K since no emulsification was observed (Fig. 4C).

**Figure 5:**
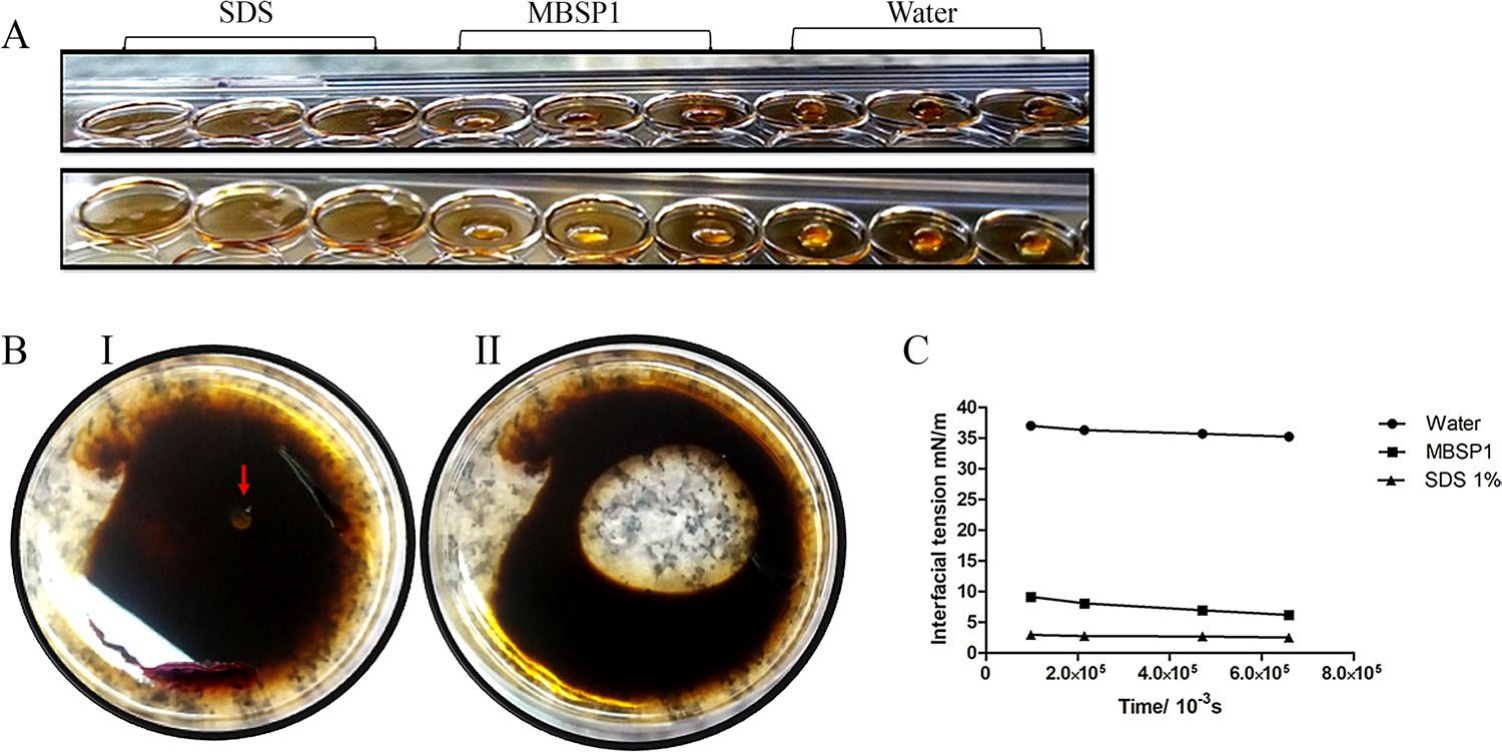
Biosurfactant activity of MBSP1 obtained by acid precipitation: (**A**) Drop collapse: a triplicate test evidencing collapsed drop in oil of both SDS positive control and biosurfactant. The water kept the formation of drops intact. (**B**) Oil dispersion test: I- MBSP1 added to plate (red arrow); II-Dispersion oil forming a halo caused by presence of MBSP1. (**C**) Interfacial tension. Mean values of interfacial tension of water, MBSP1 and synthetic surfactant SDS 1% against petroleum.

MBSP1 presented positive results for drop collapse (Fig. 5A) and oil dispersion assays (Fig. 5B). Furthermore, it significantly reduced tension interfacial against petroleum (*p* < 0.05), similar to synthetic surfactant SDS 1%. MBSP1 presented a median value of 6 N/m and synthetic surfactant SDS 1% showed an average of 2.5 N/m for interfacial tension, whereas water presented an average value of 35 N/m (Fig. 5C).

### Stability of biosurfactant

**Figure 6:**
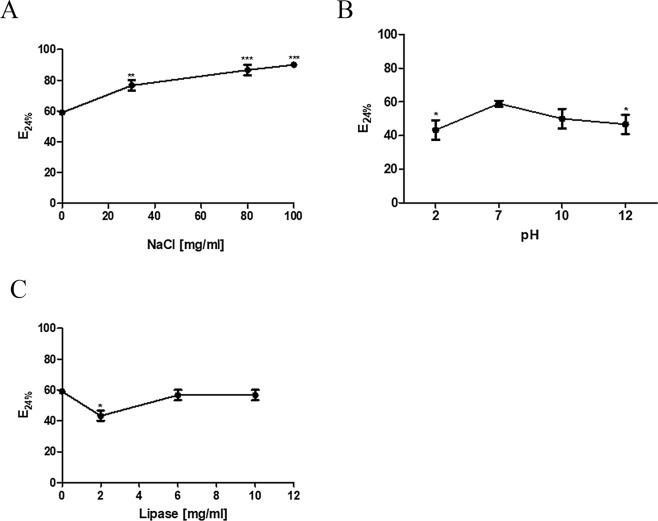
Stability of MBSP1 obtained by acid precipitation measured by emulsification test using kerosene as a substrate. (**A**) MBSP1 exposed to different concentrations of salt. (**B**) Treatment with different pH. (**C**) Treatment with lipase. Data were analysed using two‐way ANOVA followed by Dunnett’s test (*p < 0.05 to ***p < 0.0001).

The results showed that biosurfactant activity is stable in a wide range of salt concentrations and pH (Fig. 6A,B, respectively). Furthermore, an increase in the emulsification index was observed at higher salt concentrations. The biosurfactant was also tested in the presence of lipase and protease. The emulsification was not significantly affected by lipase treatment (Fig. 6C). However, no emulsifying activity was observed after the treatment of MBSP1 with proteinase K (Supplementary Fig. S3A). In addition, the emulsion was stable at high temperatures, since it was heated to 100 °C and little change was observed (Supplementary Fig. S3B).

### Hydrocarbons degradation potential

**Figure 7:**
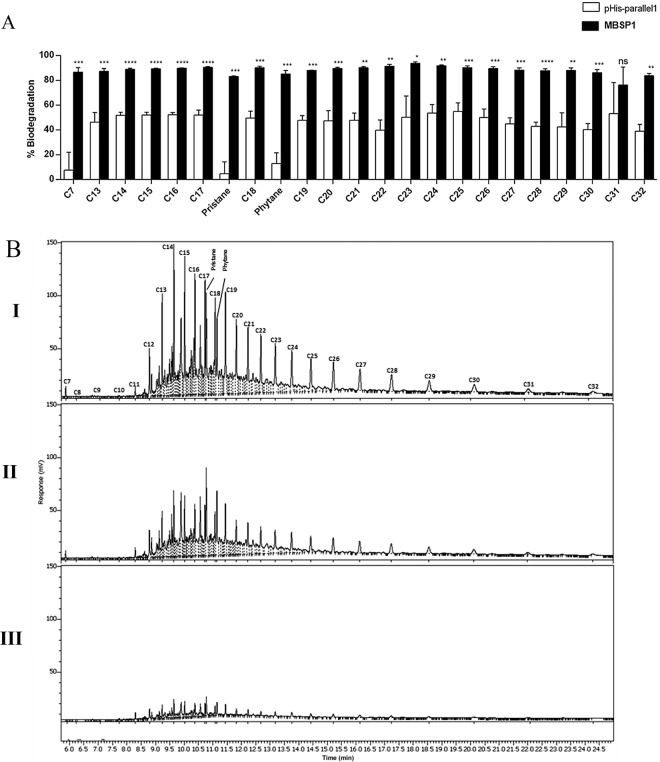
Aliphatic hydrocarbons degradation by MBPS1 clone and empty pHis-parallel1. (**A**) Comparison of percentage of aliphatic hydrocarbon biodegradation between MBSP1 and empty vector pHis-parallel1. Microorganisms were incubated in sterile BH medium contaminated with 1% (wt./v) aliphatic hydrocarbons (C7 to C32, pristane and phytane) as the source of carbon and energy, for 7 d at 30 °C and 200 × g. Data are shown as the mean ± RSD (%) from three biological replicates. Normalised data based on the negative control; unpaired Student’s t-test was performed (*p < 0.05 to ****p < 0.00001). (**B**) Aliphatic hydrocarbon degradation by I- Negative control (BH + crude petroleum); II- Empty pHis-parallel1; III-MBSP1.

To assess the hydrocarbon assimilation potential, the microbial growth behaviours of *E. coli Rosetta* ^TM^ (DE3) strain cells carrying MBSP1 clone and the empty pHis-parallel1 expression vector in BH cultures containing 1% crude oil were evaluated. The assay was monitored for 7 d, and both strains demonstrated the ability to grow in the conditions used. The pHis-parallel1 empty vector and the clone MBSP1 displayed positive results for the degradation of a wide range of aliphatic hydrocarbons (Fig. 7). The most efficient alkane and isoprenoids (pristane and phytane) degradation occurred for the MBSP1 clone (above 80%).

In contrast, the mineralisation of alkanes with C13-C32 demonstrated a decreased metabolic capacity (around 50%) by cells transformed with pHis-parallel1. This strain also reached the lowest degradation percentages for the C7, pristane and phytane chains (below 15%).

## Discussion

The MBSP1 sequence showed a high similarity to hypothetical proteins^25,26^ of the family *Halobacteriaceae*, some being identified as biosurfactant-producing species^27^. Bootstrap analysis demonstrated that the MBSP1 sequence was more comparable to *H. lucentense*. *H. lucentense* grows in a wide range of salinities (10% to 30%), and at a temperature and pH of 37 °C and 7.5, respectively^28^. This species can consume hydrocarbons such as crude oil, n-octadecane, and phenanthrene^29^, but there are no reports on the production of biosurfactants by *H. lucentense*. However, other species of the genus *Haloferax* were described as biosurfactant producers.

Djeridi *et al*.^30^ demonstrated that *Haloferax* sp. MSNC14 consumes different hydrocarbons and produces biosurfactant. However, the chemical nature of this biosurfactant was not identified. In addition, other species of *Halobacterium* were described as biosurfactant producers. Analysing the chromatographic behaviour of surfactants obtained from *Halovivax* (strain A21) and *Haloarcula* (strain D21), Kebbouche-Gana *et al*.^27^ revealed glycoproteins as the probable surfactant produced by D21, while A21 produced peptidoglycolipids (e.g., glycoprotein, glycolipid, or lipopeptide). Despite these descriptions of biosurfactant production in Halobacterium, the genes and pathways involved in the biosurfactant syntheses remain unknown. Therefore, in this study we described the first gene in the Archaea domain capable of surfactant production.

Additionally, PredictProtein software results indicated structural homology of MSBP1 with rubredoxin-like protein, but with low confidence values. Rubredoxin is a small protein with an active iron-sulphur site that is involved in the oxidation of alkanes in bacteria. Metabolic pathways were best characterised in *Pseudomonas putida* (*Oleovorans*) GPO1^31^, and is capable of oxidising C5 to C12 alkanes to 1-alkanols. Smits *et al*. reported that the alkane hydroxylase system is composed of 3 components: alkane hydroxylase (AlkB), rubredoxin (AlkG), and rubredoxin reductase (AlkT)^32^.

The physicochemical properties of the amino acid sequence revealed a transmembrane helix located at the 95^th^ residue to the 112^th^, with a size of 18 amino acid residues-long.

Surfactin, for example, forms ion-conducting channels in bacterial lipid membranes, akin to that of detergents^33^. Surfactin, as one of the most effective biosurfactants, is capable of destabilizing membranes that disrupt its integrity and permeability by forming pores^34^.

The prediction of MBSP1 subcellular localisation, considering the Archaea domain, resulted in secretion (100% confidence), with the MBSP1 protein being recovered in the supernatant. This is an interesting result since no canonical localisation domain was found in this sequence for expression in *E. coli*.

MBSP1 protein was detected with an approximate molecular weight of 20 kDa after induction with IPTG. Protein expression was also observed in the cell-free supernatant at different induction times (4 h and 18 h) and in the absence of IPTG, indicating that this protein is secreted. The molecular weight observed was different from the expected weight of approximately 32 kDa. This can be explained by the cleavage of a possible signal peptide (yet unknown). Such cleavage may lead to the release of the protein to the extracellular medium or by the disordered regions of the protein, which may be causing a differential migration in polyacrylamide gel^35^. Moreover, the structure predicted protein revealed several disordered regions.

Biosurfactant activity was positive in cell-free supernatants, purified protein, and surfactant obtained by acid precipitation from MBSP1 clone cultures. Therefore, MBSP1 is a potential candidate for use in a variety of biotechnological and industrial applications. According to Gudiña and collaborators, a broad-spectrum of emulsifying activity is essential for the use of a bioemulsifier in industrial processes, which includes different mixtures of hydrophobic compounds^36^.

In general, for an emulsion to be considered effective, the emulsification index should be higher than 40%^37^. MBSP1 presented satisfactory results (>50%), and the kerosene emulsion was stable for more than one year. Biosurfactants secreted to the extracellular environment emulsify oily compounds, increase their bioavailability, accelerate their metabolism, and mediate ecological interactions with other organisms by quorum sense regulation^38^. Interestingly, MBSP1 is an Archaea protein secreted by the *Escherichia coli* host strain. Further elucidation of this mechanism may lead to the production of other recombinant proteins that can be secreted by *E. coli*. This may facilitate the purification of more proteins with biotechnological applications.

MBSP1 differs from most biosurfactants such as glycolipids, glycopeptides, lipopeptides, and lipopolysaccharides^6^ since it is active as a single peptide. MBSP1 has surfactant functions: for example, stabilising an emulsion and reducing interfacial tension, which may be useful for biotechnological applications. The large-scale production of biosurfactants remains a challenge due to several limitations, which include dependence on complex genetic systems as operons, non-ribosomal peptide synthetases, and/or multiproteic assembly complexes. Furthermore, the large-scale biosurfactant production also depends on raw materials and adequate substrates; the availability of surfactant-producing microorganisms; adequate industrial fermenters; purification processes; biosurfactant properties; and production yields^7,8^. The characteristics presented by MBSP1 point toward the potential of large-scale biosurfactant production, thus overcoming existing limitations for the biosurfactants described so far.

The stability of biosurfactants was tested at high temperatures, under proteolytic conditions, and under different concentrations of salt and pH. A small number of bacterial species have been described as active biosurfactant producers under extreme conditions^39^. To thrive in harsh environments, microorganisms produce enzymes and metabolites that are functional under the prevailing conditions of their surroundings. MBSP1 remains active over a wide range of temperatures, pH, and salinity, which may contribute to its adaptation to extreme environmental conditions. MBSP1 was identified in a metagenomic library derived from an environment without oil contamination, differing from most biosurfactant prospective studies, which are generally performed in marine or terrestrial environments with a history of oil contamination.

When compared to synthetic surfactants, biosurfactants are generally more effective at a wide range of salinity and temperature. Temperature and saline concentration are key parameters that affect emulsifying activity in advanced oil recovery processes. The stability of MBSP1 at different salt concentrations is in accordance with soil characteristics from which this gene was identified. Thermostability is a critical property for various industrial applications of biosurfactants. Furthermore, salinity influences dispersant activity, i.e., higher salinities tend to favour the action of dispersants, suggesting that in real situations of oil spills in marine environments, dispersants would perform even better than what was observed^40^.

In addition, bacteria transformed with the empty vector pHis-parallell and clone MBSP1 presented degradability of aliphatic hydrocarbons. However, the results showed that the clone MBSP1 demonstrated an increase in degradation activity since a higher percentage of hydrocarbon degradation was observed. This increase may be attributed to the MBSP1 biosurfactant activity. In fact, Nievas *et. al*., described that the addition of biosurfactants caused an increase in the biodegradation of hydrocarbons through the mechanisms of mobilisation, solubilisation, or emulsification^41^. The increase of the biodegradation (above 60%) of the phytane and pristane compounds by MBSP1 clone reinforces this hypothesis because they are isoprenoid alkanes, which are extremely resistant to biodegradation due to the presence of molecular branching^42^.

In general, 20 to 40% of the sequences generated in metagenome projects are classified as hypothetical genes, due to the lack of similarity with known genes. In this context, functional screening has been useful for the identification of these new genes. Here, we identified and characterised a hypothetical protein that showed surfactant properties, being the first of its kind described in Archaea or Bacteria domains. We described a single gene that codifies a protein with interesting surfactant properties that can be produced in host cells such as *E. coli* without dependence on substrate, which reduces some limitations of large-scale production of biosurfactants, indicating its potential for the development of biotechnological products.

## Material and Methods

### Construction of metagenomic library and functional screening

Soil samples were collected from Jundiaí Riverbanks (Natal, Brazil), which were characterised by high salinity (description of collecting point is in Supplementary Table S1). An overview of the steps and procedures used in this study are provided in the Supplementary Fig. S6. Collection was performed using sterile tubes and spatulas, where environmental DNA (eDNA) was extracted using the commercial FastDNA^TM^ SPIN Kit for Soil (Qbiogene, Inc., Carlsbad, CA, USA) from 10 g of soil. DNA fragments (1–3 kB), obtained by sonication were inserted into pBC phagemid vectors having *E. coli* (strain DH10B) as the host strain. Strains and plasmids used in this study are listed in Table 2. A functional screening was performed to detect the presence of genes with activity in oil degradation and biosurfactant production. For the oil degradation assay, 10 µL of the culture from each clone was transferred to 96-well plates (with lid) containing 180 µL of LB medium with 25 µg/mL chloramphenicol. After a drop of light Arabic oil was added, the test plate was incubated for 15 d at 30 °C. The oil aspect was observed daily. Clones showing oil degradation were replicated in 24-well plates (with lid) containing 1.8 mL of LB medium with 25 µg/mL chloramphenicol, and the oil degradation assay was repeated. Plasmid DNA extraction was performed from clones that presented positive degradation results. Each clone was retransformed into the DH10B strain, and the oil degradation assay was repeated for confirmation. The DH10B strain containing the empty plasmid was used as a negative control. Positive clones that showed confirmed degradation activity were tested for biosurfactant production. Tests performed were: drop collapse^43^, emulsification assay^44^, and oil dispersion assay^45^. The clones with the best results were sequenced (11 clones in total) using DYEnamic ET Dye Terminator Cycle Sequencing Kit for MegaBACE^TM^ 500 (Amersham Biosciences Corp., Little Chalfont, UK) following manufacturer’s instructions. In this study, we described the execution of functional assays with one ORF identified in one clone (named 3C6) that showed positive results for all tests.

**Table 2:** Strains and plasmids used in this study.

Strains	Genotype/Description
DH10B (recA^−^)	F−endA1 *rec*A1 *gal*E15 *gal*K16 nupGrpsLΔ*lac*X74 Φ80l*ac*ZΔM15 *ara*D139 Δ(ara,leu)7697 *mcr*AΔ(mrr-hsdRMS-mcrBC) λ−
DH5α	F– Φ80*lac*ZΔM15 Δ(*lac*ZYA-*arg*F) U169 *rec*A1 *end*A1 *hsd*R17 (rK–, mK+) *pho*A *sup*E44 λ– *thi*–1 *gyr*A96 *rel*A1
*Rosetta*^TM^ (DE3)	*E. coli* strain used for protein expression; F^−^ *omp*T hsdS_B_ (r_B_^−^ m_B_^−^) *gal-dcm* (DE3) pRARE (Cam^R^)
**Plasmids**	
pBC SK	Cloning vector, Cam^r^
pCR-Blunt II-TOPO	Cloning vector, Kan^r^
pHis-parallel1	Vector for *in vitro* expression with T7 promoter, adds GB1 solubility tag; IPTG inducible; restriction enzyme cloning; Amp^r^

### ORF identification and sequence analysis

The predicted ORF sequences present in clone 3C6 were made using the ORF finder program, available online through the National Center for Biotechnology Information (NCBI) website (https://ww.ncbi.nlm.nih.gov/orffinder/) accessed in February 2017. The characterised nucleotide sequence in this study was deposited in the GenBank database under the Accession Number MK165391. Molecular weight and isoelectric point (pI) were predicted using the ExPASy Compute pI/Mw tool (http://web.expasy.org/compute_pi/). The obtained sequence was submitted to BLAST, and homologs were selected (https://blast.ncbi.nlm.nih.gov/Blast.cgi) using the non-redundant (nr) protein database. Sequences of the predicted ORF and its homologs were aligned in the CLUSTAL Omega program (Conway Institute, UCD. Belfield, Dublin 4, Ireland)^46^ and phylogenetic trees were generated by molecular evolutionary genetics analysis (MEGA) 7 software (Penn State University, University Park, PA, USA)^47^. Methods used to obtain trees were Neighbour-Joining (NJ), maximum likelihood, and maximum parsimony, all with 1000 bootstrap values. The amino acid sequence was submitted to the automatic online service, PredictProtein (PP) software (https://www.predictprotein.org/), and the protein structure prediction was submitted to the web-based service for protein structure prediction, Phyre2 software (http://www.sbg.bio.ic.ac.uk/phyre2/html/page.cgi?id = index). The identification of conserved synteny regions containing 3C6 orthologs was performed by Absynte (Archaeal and Bacterial Synteny Explorer), a web-based service designed to display local syntenies in completely sequenced prokaryotic chromosomes (http://archaea.u-psud.fr/absynte/). The Predictor of Natural Disordered Regions (http://www.pondr.com/) was utilised for the amino acid sequence, which considers a residue as disordered if its value exceeds or matches a threshold of 0.5. Peptide 2.0 (https://www.peptide2.com/N_peptide_hydrophobicity_hydrophilicity.php) was performed for verifying the peptide’s hydrophobicity.

### Sub-cloning of metagenomic ORF

Specific primers were sequenced, containing restriction sites for the BsaI and HindIII enzymes added to their 5′ regions (Table 3). The amplicon was initially cloned into a pCR®-Blunt vector (Invitrogen Corp., Carlsbad, CA, USA) and transformed into *E. coli* (strain DH5α). Enzymatic digestion with EcoRI (Biolabs, Cambridge, MA, USA) was used for cloning confirmation. The ORF of interest was excised from the cloning vector with the two enzymes, BsaI and HindIII. Then, the ORF of interest was sub-cloned into the pHis-parallel1 expression vector previously linearised with NcoI and XhoI enzymes to generate sequence ends compatible with the 3C6 ORF^48^. Ligation of inserts into the vector (3C6 + pHis-paralle1) was obtained through heat-shock, which transformed the clone into the *E. coli* strain named *Rosetta™* (DE3). *Rosetta™* (DE3) was used as the heterologous expression system. The sub-cloned ORF was named MBSP1 (metagenomic biosurfactant protein 1).

**Table 3:** Sequence of oligonucleotides and parameters used for amplification of gene coding for ORF 3C6.

Protein	Restriction Enzyme (Sequence 5′−3′)	Oligonucleotides	MT*	Fragment size
ORF3C6	Foward_BsaI GGTCTCCCATGAGTGATCAATATCT		62,9 °C	897
	pb Reverse_HindIII AAGCTTTTAAGTCGAGTCCTGACCC		64,6 °C	

*MT = Melting Temperature.

### Recombinant protein expression

*Rosetta*^TM^ (DE3) competent cells carrying the MBSP1 clone and the empty pHis-parallel1 vector were grown in 400 mL lysogeny broth (LB) medium containing 100 μg/mL ampicillin and 34 μg/mL chloramphenicol at 37 °C. Cells were grown to OD_600nm_ of approximately 0.45. Protein expression was induced with 1 mM isopropyl-β-D-1-thiogalactopyranoside (IPTG) for 18 h at 37 °C. The cell extract was collected by centrifugation at 15,000 × g for 20 min and resuspended in 1X PBS. Cell lysis was performed by adding lysis buffer (0.5 M NaCl; 10% Glycerol; 20 mM Hepes, pH 7.5; 5 mM Imidazole, pH 7.5; and 2 mM β-mercaptoethanol), 1 mM of the protease inhibitor phenylmethylsulfonyl fluoride (PMSF), 1 mg/mL lysozyme, 0.5% of 10% Triton X-100, and 150 U of Novagen^TM^ Benzonase^®^ Nuclease (10 KU − 25 U/μL) (FischerScientific, San Diego, CA, USA). The sample was kept on ice for 1 h and periodically inverted. At the end of this step, the sample was sonicated until loss of viscosity. Thereafter, each sample was centrifuged at 20,000 × g for 45 min at 4 °C. The supernatant was collected in a new pre-frozen tube. Samples were then visualised by 12% polyacrylamide gel electrophoresis (SDS-PAGE).

Detection of His-tagged fusion protein was performed using InVision™ His-tag In-gel Stain (Invitrogen Corp., Carlsbad, CA, USA). This method is sensitive, highly specific, and allows for direct visualisation of bands of the His-tagged fusion protein on a polyacrylamide gel after electrophoresis. Ni^2+^ conjugated fluorescent dye was utilised, which binds with a high affinity to histidine residues providing a clear and specific visualisation of the His-tagged protein. After electrophoresis, proteins were fixed onto the gel, followed by staining according to the manufacturer’s instructions. His-tagged protein was visualised in the ChemiDoc™ MP System (Bio-Rad, Hercules, CA, USA).

Purification of the recombinant protein was performed by affinity chromatography using the HisTrap^TM^ column (GE Healthcare, Chicago, IL, USA). In total, 20 mL of Buffer A (800 mM NaCl; 20 mM Tris-HCl, pH 8.0; 5 mM Imidazole; 2 mM β-Mercaptoethanol; and 10% Glycerol) was used to equilibrate the column. For binding the target protein to the column, the entire volume of protein extract was passed through the column. Column lavage was performed using 50 mL Buffer A and 10% Buffer B (800 mM NaCl; 20 mM Tris-HCl, pH 8.0; 300 mM Imidazole; 2 mM β-Mercaptoethanol; and 10% Glycerol). In total, 100% of Buffer B was used to elute the protein. Finally, all steps of the purification process were applied to a 12% acrylamide gel.

The biosurfactant produced by clone MBSP1 was partially purified by acid precipitation according to the method described by Vater *et al*.^49^ with minor modifications. 50 mL aliquots of bacterial culture were centrifuged in 50 mL conical tubes at 20,000 × g for 20 min at 4 °C for the removal of cells. Then, the pH of the supernatant was adjusted to 2.0 by the addition of 6.0 mol.L^−1^ of HCl and maintained at 4 °C for 18 h. The sample was centrifuged at 20,000 × g for 20 min; thereby, the supernatant was discarded, and the biosurfactant was eluted in water. The addition of 1.0 mol.L^−1^ NaOH enhanced solubilisation.

Salting-out was performed for precipitation of the biosurfactant by adding ammonium sulfate. Three different fractions were tested: 0–30%, 30–60%, and 60–90%. Initially, 8.8 g of ammonium sulfate was added to the cell-free supernatant for fraction 0–30%, and left overnight for precipitation. The sample was centrifuged at 20,000 × g for 20 min at 4 °C, and the precipitate was resuspended in water. Eluted proteins were dialysed in 200 mL of buffer (800 mM NaCl; 20 mM Tris-HCl, pH 8.0; 5 mM Imidazole; 2 mM β-Mercaptoethanol; and 10% Glycerol) at 4 °C for 16 h in SnakeSkin^TM^ Dialysis Tubing (68100) (ThermoScientific, San Diego, CA, USA) of 10 kDa molecular weight cut-off (MWCO). This procedure was repeated for the remaining fractions where only the amount of ammonium sulfate added was changed. For the fraction 30–60%, 9.9 g was added, and for the fraction 60–90%, 11.35 g of ammonium sulfate was added. The recombinant protein obtained was purified as previously described and observed on a 12% polyacrylamide denaturing gel (SDS-PAGE).

### Biosurfactant activity

The emulsification index (E_24%_) of culture samples were determined by adding 2 mL of a hydrocarbon (kerosene, diesel, hexane, hexadecane, toluene, and xylene) to the same amount of supernatant (of clone MBSP1 and empty vector). Mixing was accomplished by vortex for 2 min, followed by a 24 h rest period. E_24%_ was determined as the height of the emulsion layer divided by the total height and multiplied by 100^44^. The assay was performed in duplicates.

Cell-free supernatants were incubated with different concentrations of proteinase K (5, 10, and 20 mg/mL) at 37 °C for 10 min. Then, an emulsification assay was performed using kerosene as the hydrophobic substrate.

The oil dispersion test, the drop collapse test, and evaluation of interfacial tension were performed with the precipitated biosurfactant. For the oil dispersion test, 1 mL of oil was added to the surface of 40 mL of distilled water in a Petri dish, forming a thin layer of oil. Then, 10 μL of biosurfactant was gently added at the centre of the oil layer^50^. The drop collapse test described by Jain *et al*., was performed on the cover of a 96-well plate^43^. To the halos were added 2 μL of oil, which was allowed to stand for 24 h at 25 °C for stabilisation. On the following day, 5 μL of biosurfactant was added, and the drop form was inspected after 1 min. The assay was performed in triplicates. SDS (20%) and water were used as positive and negative controls, respectively. Interfacial tension was evaluated by the Drop Volume Tensiometer, model DVT50 (Kruss Scientific, Hamburg, Germany) using the rising drop method, in which the force between the liquid containing surfactant (bulk phase) and oil droplet formed in the dispense phase was evaluated. The test was performed using 15 mL of biosurfactant in the bulk phase and petroleum in the dispense phase. The assay was performed according to the manufacturer’s instructions. SDS (1%) and water were used as positive and negative controls, respectively.

### Stability of biosurfactant

The stability of biosurfactant obtained by acid precipitation was tested to determine its emulsification ability (using kerosene as the hydrocarbon source) after several treatments. All assays were performed in a 2 mL microtube. For the thermostability test, the biosurfactant was subjected to a temperature of 100 °C for 1 h. For the halo-stability study, different concentrations of sodium chloride (30, 80, and 100 mg/ml) were added to the biosurfactant. The biosurfactant was subjected to different pH conditions (2, 7, 10, and 12) by the addition of hydrochloric acid and sodium hydroxide. To test biosurfactant proteolytic resistance, 0.1, 0.2, and 0.3 mg/ml of proteinase K were added. To analyse its lipase resistance, 2.0, 6.0, 7.0, and 10 mg/ml of this enzyme were added.

### Petroleum hydrocarbons degradation analysis

*E. coli* strain *Rosetta*^TM^ (DE3) cells carrying MBPS1 and empty pHis-parallel1 were evaluated for their biodegradation ability using crude petroleum from Brazil with predominant concentrations of aliphatic hydrocarbons (C7-C32). The microorganisms were pre-cultured in 50 mL lysogeny broth (LB) medium containing 100 μg/mL ampicillin and 34 μg/mL chloramphenicol at 37 °C. Protein expression was induced with 1 mM IPTG for 18 h at 37 °C. The cells were centrifuged (15,000 × g, 20 min, 4 °C), washed twice, and suspended (0.1 OD_600nm_) with sterile Bushnell-Haas (BH) medium (Sigma-Aldrich Corp., St. Louis, MO, USA). The cultures were inoculated in 20 mL of sterile BH medium supplemented with crude petroleum (1%) wt./v and incubated on a rotatory shaker (180 × g) for 7 d at 30 °C. Biodegradation negative controls were performed with no addition of microbial inoculum.

After 7 d of incubation, petroleum hydrocarbon fractions were subjected to a liquid-liquid extraction process. The extract was concentrated in a rotary evaporator and subjected to preparative liquid chromatography to clean up the aliphatic fraction (F1). The separation of F1 was performed in glass columns, where silica gel 60 (SiO_2_), aluminium oxide 90 (Al_2_O_3_), and sulphate chloride (Na_2_SO_4_) were used as the stationary phase; n-hexane comprised the mobile phase^51,52^. The identification of the constituents of the aliphatic fraction was based on the respective retention times of analytical standards^51^. The aliphatic hydrocarbons were analysed by Gas Chromatography with Flame Ionization Detector GC-FID on a Clarus® 600 Chromatograph Adapter (PerkinElmer, Inc., Waltham, MA, USA)^53^. Quantitative analyses were performed using the modified external standardisation method^54–56^. Biodegradation percentage was calculated based on the following Eq. (1).

1 $${B}_{p}=[({C}_{i}-{C}_{f})/{C}_{i}]\times 100$$

Where Bp refers to the biodegradation percentage at the end of incubation time; ${C}_{i}$ represents the amount of contaminant at the start of incubation; and ${C}_{f}$ represents the amount of contaminant at the end of incubation^57^.

### Statistical analysis

Statistical comparisons for the emulsification index between MBPS1 and positive control 1% SDS was done using unpaired t-test with Welch’s correction. For multiple comparisons between treatments, ANOVA followed by Dunnett’s test were used for the parametric ANOVA test applied interfacial tension assessment. For aliphatic hydrocarbons degradation assay, the unpaired Student’s t-test was used. In all tests, values of *p* < 0.05 were considered significant.

## Supplementary information

Supplementary material.
