# Supplementary material for: MBSP1: a biosurfactant protein derived from a metagenomic library with activity in oil degradation
Source: Sci Rep. 2020 Jan 28;10:1340. doi: 10.1038/s41598-020-58330-x (PMC6987170; doi:10.1038/s41598-020-58330-x)
Supplement: Supplementary file 1 — Supplementary material. [file 41598_2020_58330_MOESM1_ESM.pdf]

Supplementary Material:

MBSP1: a biosurfactant protein derived from a metagenomic library with activity in oil degradation

Sinara Carla da Silva Araújo[1]; Rita C. B. Silva-Portela[1]; Daniel Chaves de Lima[1]; Marbella Maria Bernardes da Fonsêca[1]; Wydemberg J. Araújo[1]; Uaska Bezerra da Silva[1] Amanda P. Napp[2]; Evandro Pereira[2]; Marilene H. Vainstein[2]; Lucymara Fassarella Agnez-Lima[*].

*Corresponding Author:
Dr. Lucymara Fassarella Agnez-Lima
Departamento de Biologia Celular e Genética, Centro de Biociências,
Universidade Federal do Rio Grande do Norte,
Campus Universitário, Lagoa Nova, Natal-RN,
59078-900, Brazil.
Tel. # 55.84.3211-9209, Fax # 55.84.3215-3346
E-mail: lfagnez@ufrnet.br

This file contains:

Supplementary Figures S1 to S6

Supplementary Tables S1 to S2

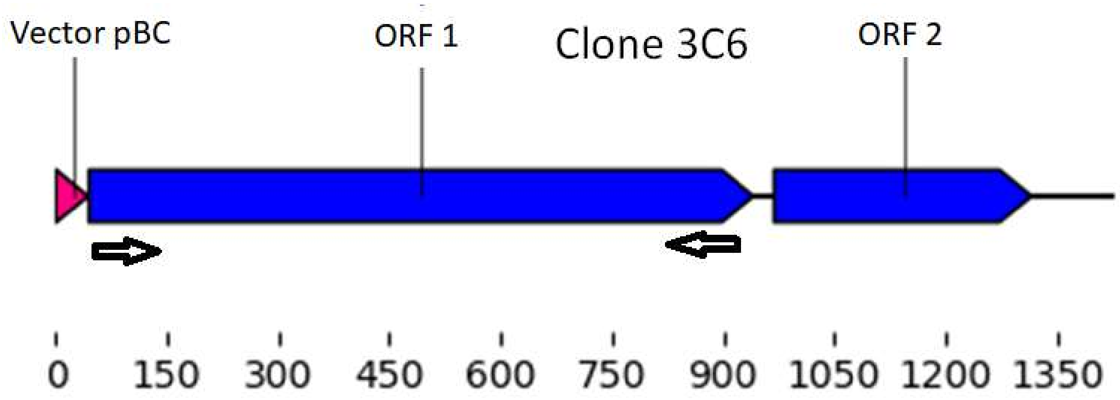

Supplementary Figure S1: ORFs identified in clone 3C6. The ORF1 contains 897 bp and was subcloned into a pHis-parallel1 expression vector in this work (⇦primers used for sequence amplification). Both ORFs show high identity with conserved hypothetical ORFs from *Halobacteriaceae* family.

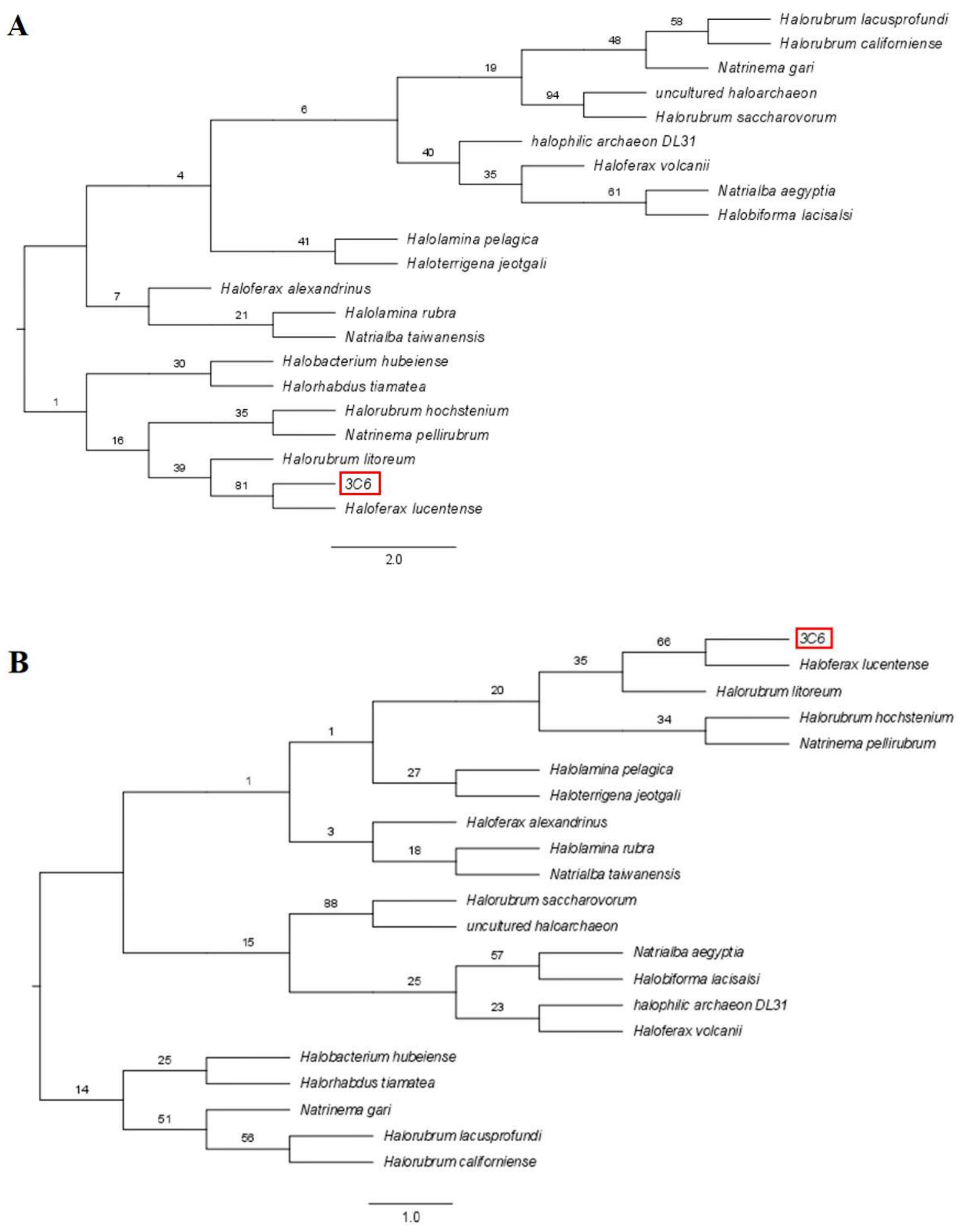

Supplementary Figure S2: Phylogenetic trees evidencing highest similarity with *Haloferax lucentense* proteins. A) Phylogenetic tree obtained from alignment by maximum parsimony; B) Phylogenetic tree obtained from alignment by maximum likelihood.

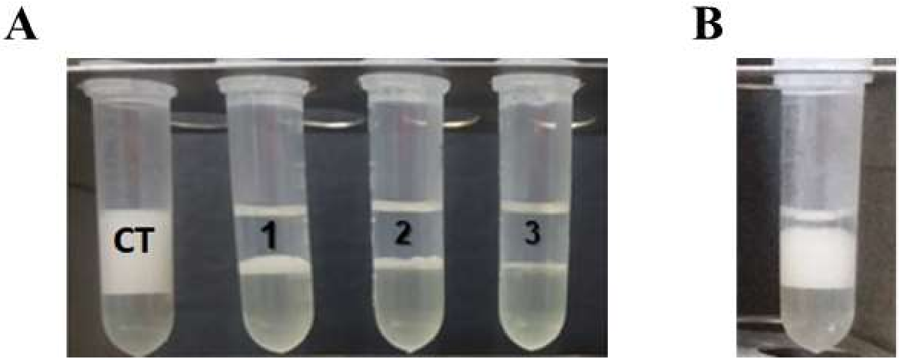

Supplementary Figure S3: Stability of MBSP1 obtained by acid precipitation measured by emulsification test using kerosene as a substrate A) Addition of proteinase-k: CT-control without addition of proteinase K; 1- 0.1 mg/ml of proteinase K; 2- 0.2 mg/ml of proteinase K; 3- 0.3 mg/ml of proteinase K. B) Emulsion with MBPS1 heated to100 °C.

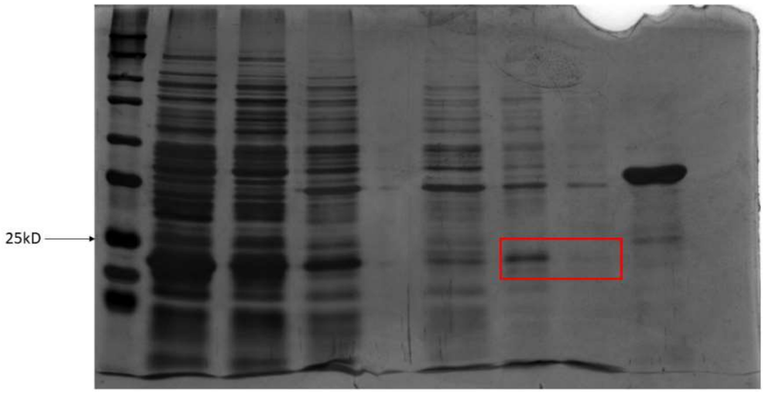

Uncropped

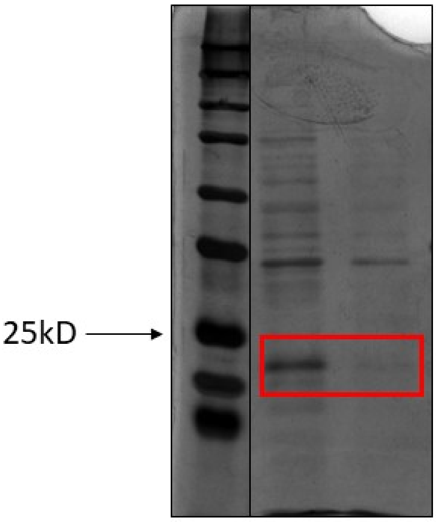

Cropped

Supplementary Figure S4: Uncropped and cropped **figure 3C**. The highlighted samples are part of the study. Additional samples are not part of this study. The black line delineates where the gel was cut.

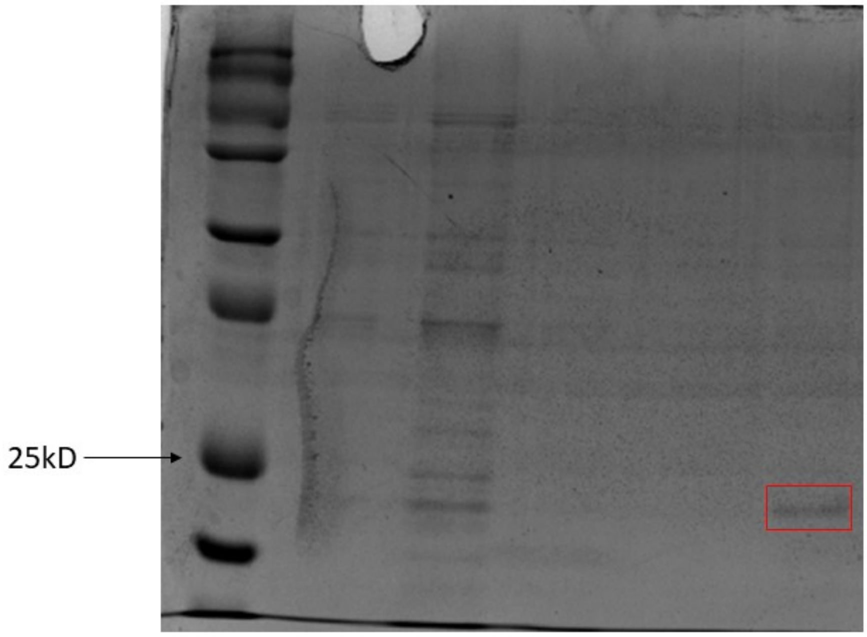

Uncropped

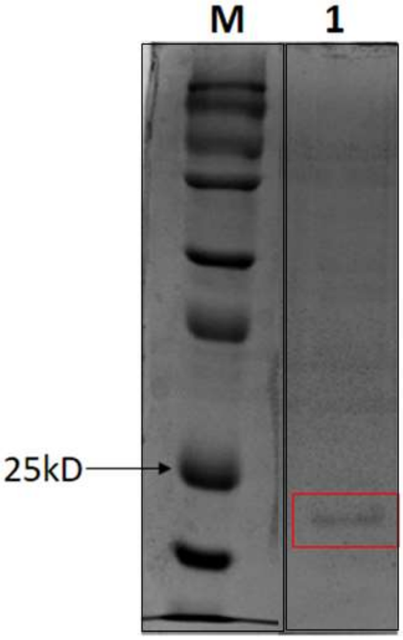

Cropped

Supplementary Figure S5: Uncropped and cropped **Figure 3D**. The highlighted sample is part of the study. Additional samples are not part of this study. The black line delineates where the gel was cut.

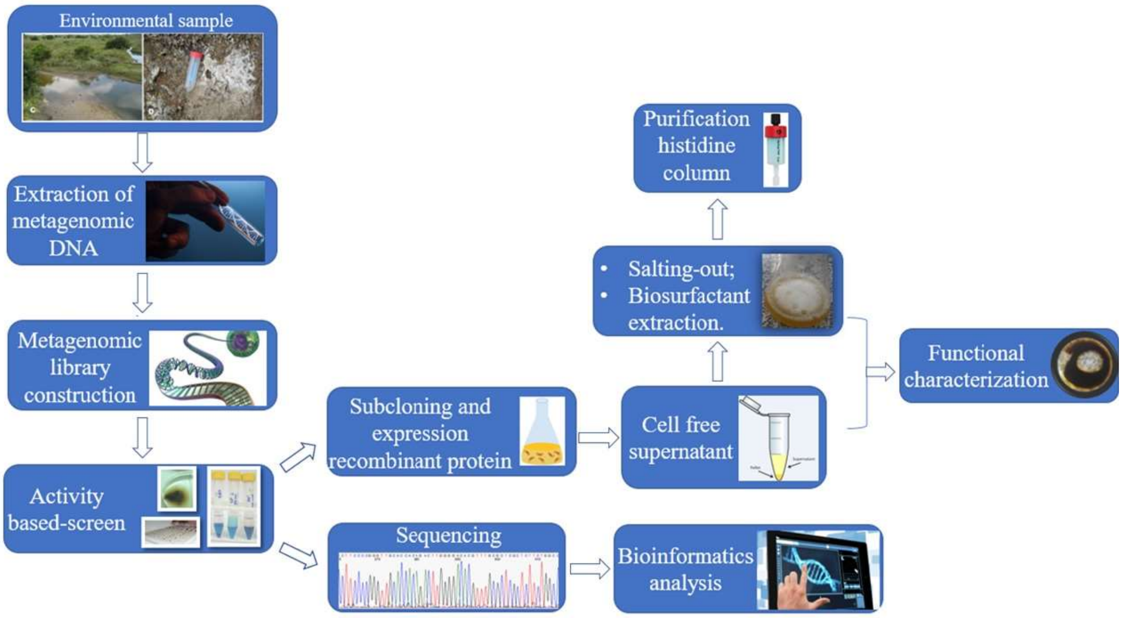

Supplementary Figure S6: Visualization of the work flow for prospecting and obtaining a clone with biosurfactant activity obtained from metagenomic library. The construction of the metagenomic library, functional screening, expression of the recombinant protein and its characterization.

Supplementary Table S1: Soil characteristics and geographical references of the area of sample collection.

Geographic reference	Conductivity ($dS.m^{-1}$)	pH	Granulometry ($g.Kg^{-1}$)			Sodium ($mg.dm^{-3}$)
			**Sand**	**Clay**	**Silt**	
**S 05° 51’ 926’’ W 35° 21° 310’’**	4,3	6,5	955	20	25	90

Supplementary Table S2: BlastP results of the 3C6 ORF.

**Description of BlastP hit (ORF3C6)**	**E-value**	**Identity**	**Accession**
hypothetical protein [*Haloferax lucentense*]	0.0	93%	WP_004062196.1
hypothetical protein [*Halorubrum trapanicum*]	0.0	91%	WP_096396384.1
hypothetical protein [*Natrialba taiwanensis*]	0.0	90%	WP_083867309.1
hypothetical protein [*Haloterrigena jeotgali*]	0.0	92%	WP_084158367.1
hypothetical protein [*Halorubrum* sp. SD683]	0.0	94%	WP_086215034.1
hypothetical protein [*Halobacterium hubeiense*]	0.0	89%	WP_059058917.1
hypothetical protein [*Halobellus clavatus*]	0.0	88%	WP_089767349.1
hypothetical protein [*Haloarchaeobius iranensis*]	6e-180	88%	WP_089736699.1
hypothetical protein [*Halolamina pelagica*]	1e-179	88%	WP_074879642.1
Conserved hypothetical protein [*Halorhabdus tiamatea* SARL4B]	2e-179	91%	CCQ35110.1
